# A taxonomic revision helps to clarify differences between the Atlantic invasive *Ptilohyale littoralis* and the Mediterranean endemic *Parhyale plumicornis* (Crustacea, Amphipoda)

**DOI:** 10.3897/zookeys.754.22884

**Published:** 2018-04-30

**Authors:** Sabrina Lo Brutto, Davide Iaciofano

**Affiliations:** 1 Department STeBiCeF, Section of Animal Biology, via Archirafi 18, University of Palermo, Palermo, Italy

**Keywords:** Atlantic, Hyalidae, *Invasive species*, Mediterranean Sea, *Parhyale plumicornis*, *Ptilohyale littoralis*

## Abstract

*Ptilohyale explorator* (formerly *Parhyale explorator*), described by Arresti (1989), can be considered to be a synonym of west-Atlantic *Ptilohyale littoralis* (Stimpson, 1853), based on morphological observations of paratypes and specimens recently collected in the type locality of *Ptilohyale explorator*. The first collections of *Ptilohyale littoralis*, from the eastern Atlantic were from the port of Rotterdam (The Netherlands) in 2009 and later in Wimereux, Opal Coast (France) in 2014; however, the synonymy of *Ptilohyale explorator* with *Ptilohyale littoralis* backdates to the first European record of *Ptilohyale littoralis* in 1985 at La Vigne, Bay of Arcachon (France). This indicates that *Ptilohyale littoralis* has been established along European Atlantic coast for many years.

An assessment of the nominal valid species belonging to the genus *Ptilohyale* was carried out and a comparison between the Atlantic *Ptilohyale littoralis* and the very similar Mediterranean hyalid species, *Parhyale plumicornis*, is presented based on morphological features and distribution. Due to the invasive ability of *Ptilohyale littoralis*, a comparison between the two species is necessary.

## Introduction

*Ptilohyale explorator* (formerly *Parhyale explorator*) was described by Arresti (1989) from La Vigne, Bay of Arcachon, France. He collected eleven male and three female specimens in the intertidal zone, on the sand of a semi-enclosed beach under stones, in July 22, 1985; following Barnard’s (Barnard 1979: 120) “Key to the Species of Parhyale and Parallorchestes”, he established that the specimens sampled showed a feature that was not included in the key, i.e., the presence of long tufts of plumose setae in antenna II starting at the 5^th^ peduncular segment. However, as Barnard’s taxonomic key (Barnard 1979) had omitted some hyalid species already described at that time, Arresti did not take into consideration some preceding descriptions (listed in Table 1) that could fit with his collected specimens.

**Table 1.** List of *Parhyale* and *Ptilohyale* species excluded by Barnard’s taxonomy key (Barnard 1979: 120) and by Arresti (1989), here named according to Lowry (2010) and Lowry et al. (2010).

*Ptilohyale littoralis* (Stimpson, 1853) – formerly *Allorchestes littoralis*
*Ptilohyale plumulosus* (Stimpson, 1857) – formerly *Allorchestes plumulosus*
*Ptilohyale crassicornis* (Haswell, 1879) – formerly *Allorchestes crassicornis*
*Parhyale inyacka* KH Barnard, 1916
*Ptilohyale barnardi* (Chevreux, 1926) nomen dubium – formerly *Hyale barnardi*
*Ptilohyale ptilocerus* (Derzhavin, 1937) – formerly *Allorchestes ptilocerus*
*Ptilohyale tristanensis* (Macnae, 1953) nomen dubium – formerly *Allorchestes tristanensis*
*Ptilohyale iole* (JL Barnard, 1970) – formerly *Hyale iole*
*Ptilohyale barbicornis* (Hiwatari & Kajihara, 1981) – formerly *Hyale barbicornis*

As a consequence, Arresti described a new hyalid species under the name *Parhyale explorator*, and deposited eight males and two females in the laboratory of the University of the Basque Country (Spain; holotype; allotype; six males and one female paratypes), one male paratype in the Carcinology Laboratory of Natural History Museum of Paris (France), and one male and one female paratypes in the Laboratory of Dr. S. Ruffo in the Museum of Natural History of Verona (Italy).

In 2008, *Ptilohyale explorator* (Arresti 1989) was reported as a new alien species within the Mediterranean Sea (Bakir et al. 2008), but later acknowledged to be a misidentification (Bakir et al. 2013), who re-identified the samples as *Parhyale plumicornis* (Heller, 1886), an endemic Mediterranean species (Iaciofano and Lo Brutto 2017). Regrettably, the case of this erroneous identification caused a cascade-effect on successive papers and documents that reported a further non-indigenous species (NIS) within Mediterranean (Bakir et al. 2010, Christodoulou et al. 2013, Faasse 2014, Zenetos 2010, Zenetos et al. 2010), although this was not the case.

*Ptilohyale explorator* is currently considered a valid species even if some authors have already highlighted the need of further investigations, in light of its high similarity with *Ptilohyale littoralis* (Faasse 2014, Spilmont et al. 2016, Marchini and Cardeccia 2017).

To clarify the position of *Ptilohyale explorator*, here considered *species inquirenda*, the paratypes deposited at the Natural History Museum of Verona and at the Natural History Museum of Paris were examined, together with some topotypic specimens collected in the type locality of the species, Bay of Arcachon, France. Descriptions and illustrations of current species belonging to the genus *Ptilohyale* were also consulted, and it was observed that some of them were not ascribable to this genus.

## Materials and methods

The paratypes of *Parhyale explorator* (voucher number 330/P) deposited in Sandro Ruffo’s collection of the Museum of Natural History of Verona, Verona (Italy) and the *Ptilohyale explorator* paratype (voucher number MNHN-Am3957) deposited at the crustaceans collection of the Natural History Museum of Paris (MNHN), Paris (France) were examined under a stereo-microscope, and photos were produced.

Additionally, a total of 126 specimens of *Ptilohyale* sp. (84 females and 42 males) was collected in the intertidal zone associated with mussel beds (*Mytilus edulis*), from Bay of Arcachon, France (the type locality of *Ptilohyale explorator*), 43°34’N, 1°14’W (DDM), in October 2015, and fixed in 95% ethanol. Their body lengths, from tip of rostrum to apex of telson, were measured using ImageJ software after placement on graph-paper and photography (FINEPIX S1800, FUJIFILM); pencil drawings were scanned and ‘inked’ using the software Adobe Illustrator CS5. The specimens were identified as *Ptilohyale littoralis* and deposited at the Museum of Zoology of the University of Palermo (MZPA), Palermo (Italy), Voucher Number MZPA-AMPH-0024.

Descriptions of the 12 world species of the genus *Ptilohyale*, according to the World Amphipoda Database (Horton et al. 2017), were consulted and the diagnostic characters delimiting *Ptilohyale* Bousfield & Hendrycks, 2002 were verified: (1) heavily plumose (finely brush-setose) antenna II starting at the 5^th^ peduncular article (both sexes); (2) lack of a guiding robust seta on the medial face of the propodus of gnathopod I (male); (3) variously developed carpal lobe of gnathopod II (male); (4) distomedial robust seta on the peduncle of uropod I; (5) inner ramus of uropod III more or less fused to the peduncle. The subsequent generic status for each of these species was then revised.

## Results

**Figure 1.**
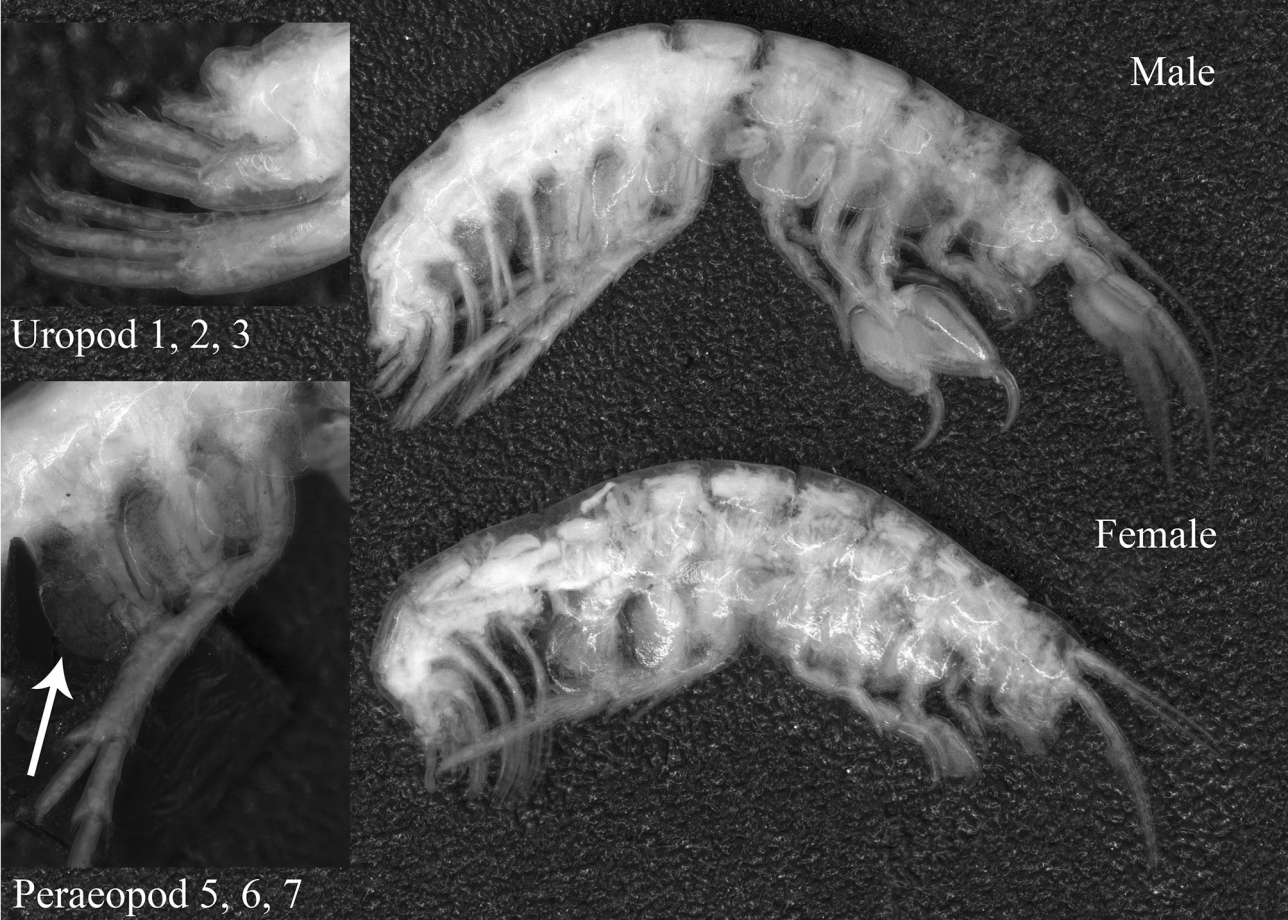
Male and female paratypes of *Parhyale explorator* (subsequently synonymised *Ptilohyale explorator*) from Ruffo’s collection (Museum of Natural History of Verona, Italy), entire samples; male peraeopods and uropods, with focus (arrow) on basipodite of peraeopod VII.

The paratypes of *Ptilohyale explorator* preserved in the Museum of Natural History of Verona were entire and in good condition for observations (Fig. 1), while the paratype stored in the Carcinology Laboratory of Natural History Museum of Paris had deteriorated and some body parts were lost (i.e., heads).

Comparison with the description of *Ptilohyale explorator* (Arresti 1989: 103–111) and the paratypes stored at the museums of Verona and Paris showed some incongruences (Table 2). The most significant difference was the absence of a strong depression on the basipodite of peraeopod VII in all paratypes, conversely to what was indicated in the description. Other diagnostic characters described by Arresti (1989) were also unlike the paratypes, including the number of plumose articles on antenna II, and the arrangement of setae on uropods I and III (see Table 2 for details).

**Table 2.** Diagnostic character states observed in the *Ptilohyale explorator* (*species inquirenda*) paratypes stored at the Museum of Natural History of Verona (Italy) and the Natural History Museum of Paris (France), and in the *Ptilohyale littoralis* sampled in the Bay of Arcachon (France); compared with Arresti’s description of *Ptilohyale explorator* and Bousfield and Hendricks’s *Ptilohyale littoralis* description. The table shows the incongruences (*) between the description of *Ptilohyale explorator* by Arresti and the deposited paratypes.

**Characters**	**Samples of *Ptilohyale littoralis* (this paper)**	**Bousfield and Hendricks’s description of *Ptilohyale littoralis***	**Paratypes of *Ptilohyale explorator* (deposited by Arresti at Museum of Verona)**	**Paratype of *Ptilohyale explorator* (deposited by Arresti at Museum of Paris)**	**Arresti’s description of *Ptilohyale explorator***
Antenna II, flagellar articles ventrally setose *	4–9	NA	8	NA	10–11
Coxal plate I	subquadrate	subquadrate	subquadrate	NA	subquadrate
Gnathopod I, basis distinct anterodistal lobe	absent	absent	absent	NA	absent
Peraeopod VII basis*	without strong depression on posterior margin	without strong depression on posterior margin	without strong depression on posterior margin	without strong depression on posterior margin	with strong depression on posterior margin
Uropod I rami spines*	3–4 outer; 1–2 inner	2–3 outer	3 outer; 2 inner	NA	6 outer; 2 inner
Uropod II rami	subequal	subequal	subequal	subequal	subequal
Uropod III apical spines*	5–9	5–6	5–6	NA	8–10

NA, not available

Following the detailed description updated by Faasse (2014) and the recent Hyalidae taxonomic key presented by Bousfield and Hendrycks (2002), the paratypes of Ruffo’s collection and all 126 specimens sampled at the Bay of Arcachon (France), were identified as specimens of *Ptilohyale littoralis*. *Ptilohyale explorator* (formerly *Parhyale*) (Arresti, 1989) can be considered synonym of *Ptilohyale littoralis* (Stimpson, 1853).

## Systematics

### Suborder SENTICAUDATA Lowry & Myers, 2013

#### Infraorder TALITRIDA Rafinesque, 1815

##### Superfamily TALITROIDEA Rafinesque, 1815

###### Family HYALIDAE Bulycheva, 1957

####### Subfamily HYALINAE Bulycheva, 1957

######## Genus *Ptilohyale* Bousfield & Hendrycks, 2002

######### Ptilohyale littoralis

Taxon classificationAnimaliaAmphipodaHyalidae

(Stimpson, 1853)

Figures 2 , 3

Allorchestes littoralis Stimpson, 1853: 49, t 3, fig. 36; Smith 1873: 556; Stebbing 1906: 595; Miner 1950: 462, pl. 148.
Hyale littoralis (Stimpson, 1853) Holmes 1905: 472, pl. 3, fig. 2; Barnard and Karaman 1991: 369.
Hyale prevosti (part) Della Valle, 1893: 519.
Hyale plumulosa (Stimpson, 1853) Bousfield 1973: 155, pl. XLIV.2; Pollock 1998: 241, fig. 15.120.
Plumulohyale plumulosa (Stimpson, 1853) Bousfield 2001: 104.
Ptilohyale littoralis (Stimpson, 1853) Bousfield and Hendrycks 2002: 103; Faasse 2014: 1.
Parhyale explorator Arresti, 1989: 101–115.
Ptilohyale explorator (Arresti, 1989) Bousfield and Hendrycks 2002: 98–99.

########## Type.

Neotype deposited in Canadian Museum of Nature Collection; voucher number CMNC 2002-0071 (Bousfield and Hendrycks 2002).

########## Type locality.

Grand Manan Island (Canada), northern eastern Atlantic coast.

########## Material examined.

One hundred and twenty-six specimens were collected at the Bay of Arcachon France (43°34’N, 1°14’W), 13October 2015; intertidally, 0 m, on the heavy substrate of the semi-closed beach (MZPA-AMPH-0024).

**Figure 2.**
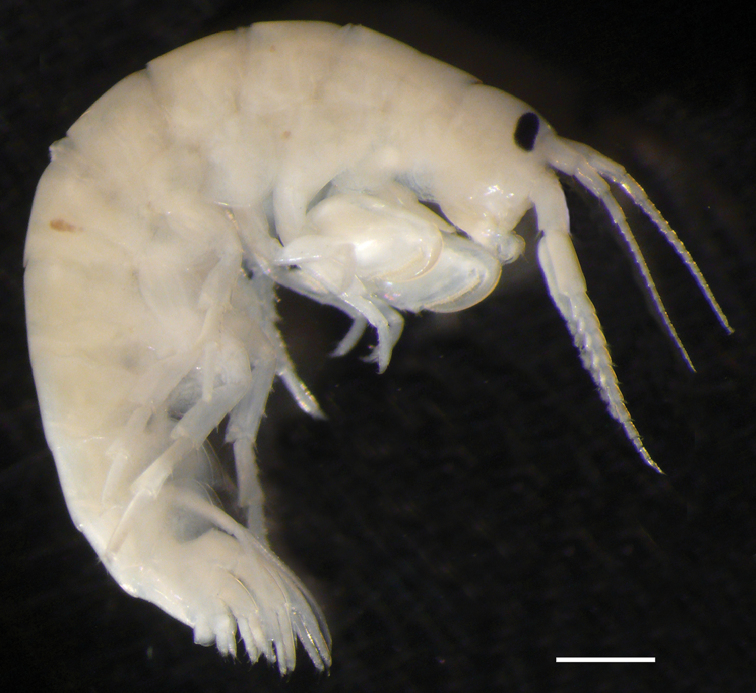
Male of *Ptilohyale littoralis*, sampled in October 2015, from Bay of Arcachon, France. Scale bar 1 mm.

**Figure 3.**
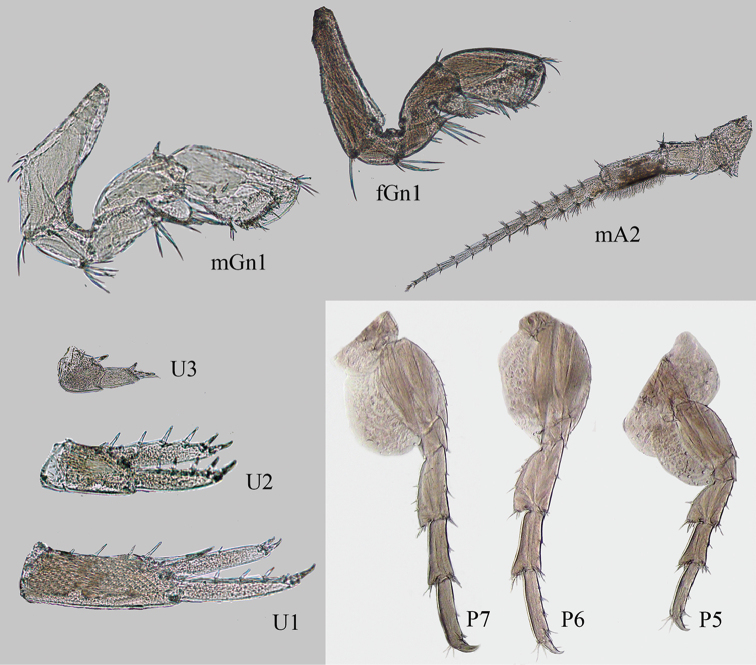
*Ptilohyale littoralis*, antenna II male (mA2), gnathopod I male (mGn1), gnathopod I female (fGn1), peraeopods V (P5), VI (P6) and VII (P7), uropods I (U1), II (U2) and III (U3).

########## Description.

***Male.*** 11.4 mm length specimen. Antenna II ventral margins of the 5^th^ peduncular article and first 4–9 flagellar articles (other one or two articles with sparse plumose setae) densely covered with plumose setae (brush setae). Palp of maxilla I with median constriction. Coxal plate I sub-quadrate with distinctive cups; Gnathopod I, basis lacking distinct anterodistal lobe (hydrodynamic lobe). Gnathopod II, carpus lobe present in juvenile male and absent on adult male. Coxal plate V posterior lobe smaller than anterior lobe; Peraeopod V, basis rounded. Peraeopod VII slender, basis rounded. Uropod I, peduncle with one distomedial robust seta; rami subequal with 3–4 robust setae on outer ramous and 1–2 robust setae on inner ramus. Uropod II, rami sub-equal in length. Uropod III, outer ramus with 5–9 apical robust setae. Telson acute. ***Female.*** Description based on a 10.6 mm length specimen. Gnathopod I, basis with anterodistal lobe.

########## Distribution.

Northern, western, and eastern Atlantic coasts; north eastern Pacific coast.

########## Remarks.

The genus *Ptilohyale* includes 12 species: *P. barbicornis* (Hiwatari & Kajihara, 1981); *P. barnardi* (Chevreux, 1925); *P. bisaeta* (Kim & Kim, 1991); *P. brevicrus* Eun et al., 2014; *P. crassicornis* (Haswell, 1879); *P. eburnea* (Krapp-Schickel, 1974); *P. explorator* (Arresti, 1989); *P. iole* (Barnard, 1970); *P. littoralis* (Stimpson, 1853); *P. plumulosus* (Stimpson, 1857); *P. ptilocerus* (Derzhavin, 1937); *P. tristanensis* (Macnae, 1953) (Bousfield and Hendrycks 2002, Eun et al. 2014, Lowry 2010). Of these, the descriptions of three of the species showed characters not ascribable to *Ptilohyale* *sensu* Bousfield & Hendrycks (2002). *Ptilohyale barnardi* (formerly *Hyale barnardi*) has brush-setae in antenna II that start at the 4^th^ peduncular article (see Chevreux 1925, Fig. 4A); *P. tristanensis* (formerly *Allorchestes tristanensis*) (see Macnae 1953, Fig. 4B) and *P. eburnea* (see Krapp-Schickel 1974, Fig. 4C), do not have brush-setae in antenna II. The absence of some diagnostic character states makes us consider *Ptilohyale barnardi*, *P. tristanensis*, and *P. eburnea* as *nomina dubia*, and we encourage further investigations.

**Figure 4.**
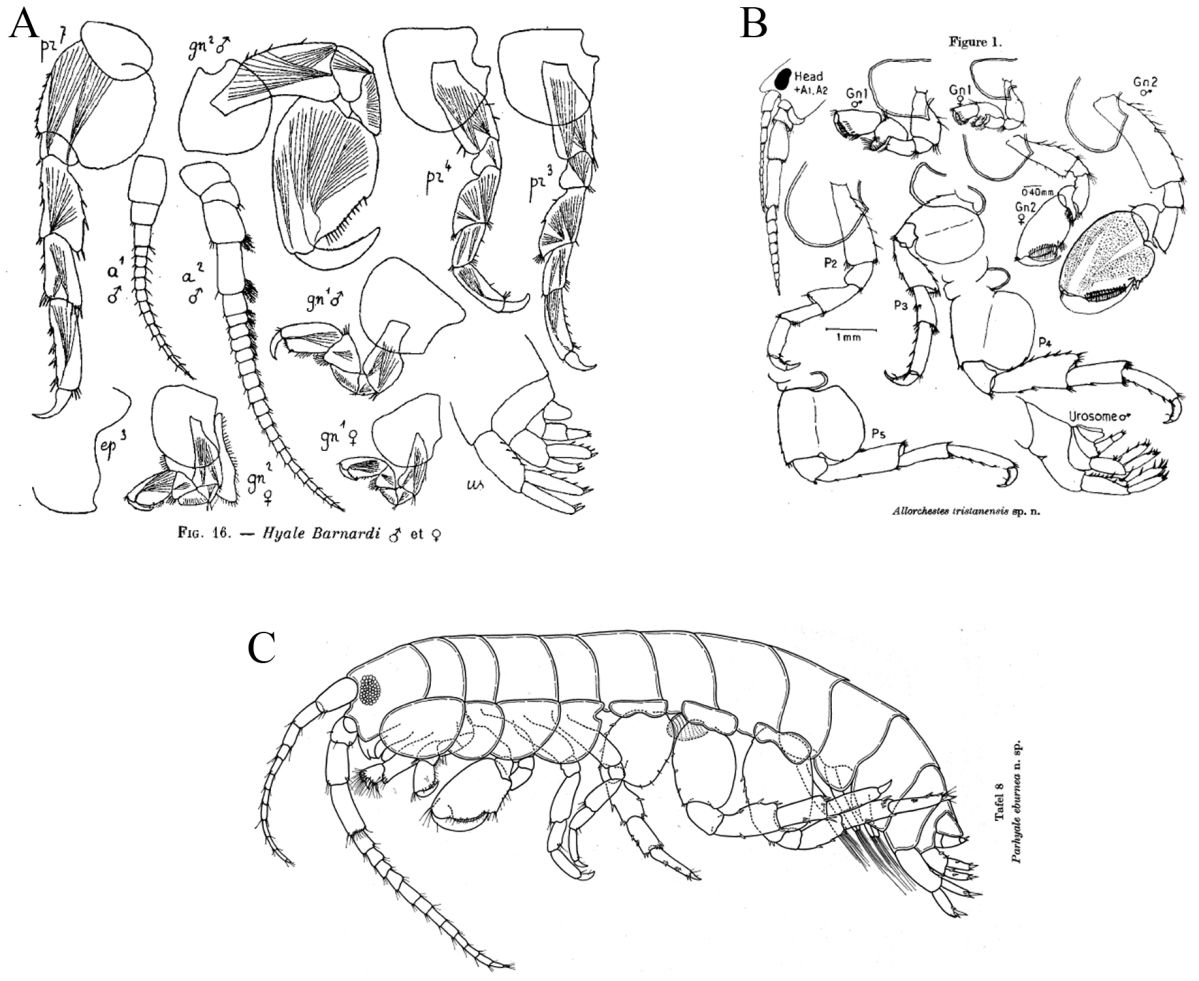
Illustrations from the literature of: **A** *Ptilohyale barnardi* (Chevreux, 1925) **B** *Ptilohyale tristanensis* (Macnae, 1953) **C** *Ptilohyale eburnea* (Krapp-Schickel, 1974).

## Discussion

*Ptilohyale* (formerly *Parhyale*) *explorator* was described by Arresti (1989) using the dichotomous key to “*Parhyale* and *Parallorchestes*” of Barnard (1979: 120); he observed that the specimens collected were not ascribable to any of the species listed therein, due to the presence of *dense elongate tufts of plumose setae ventrally on the peduncular article 5 of the antenna II* and *peduncle of uropod I with distomedial robust seta*. These characters (and others listed in Table 3) prompted Arresti to describe a new species, and to revise Barnard’s key; however, both authors had excluded some hyalid species that could be identified with Arresti’s specimens (Table 1).

**Table 3.** Characters used by Arresti (1989) for diagnosing *Parhyale explorator* (subsequently synonymised *Ptilohyale explorator*) from the other species of the genus *Parhyale*.

*Parhyale explorator*	*Parhyale eburnea* Krapp-Schickel, 1974
Uropod I with robust seta on peduncle; Rami of uropods I and II with strong dorsal setae.	Uropod I without robust seta on peduncle; Rami of uropods I and II without strong dorsal setae.
*Parhyale explorator*	*Parhyale plumicornis* (Heller, 1866)
Uropod III with only apical setae; Inner ramous of uropod III poorly defined and fused to the peduncle; Carpus of gnathopod II male with stout process.	Uropod III with apical and dorsal setae; Inner ramous of uropod III well defined and not fused to the peduncle; Carpus of gnathopod II male with evident process.
*Parhyale explorator*	*Parhyale aquilina* (Costa, 1857)
Uropod I with robust seta on peduncle.	Uropod I without robust seta on peduncle;
*Parhyale explorator*	Parhyale ? zibellina (Derzhavin, 1937)
Uropod III with only apical setae; Inner ramous of uropod III poorly defined and fused to the peduncle; Uropod I with robust seta on peduncle.	Uropod III with apical and dorsal setae; Inner ramous of uropod III well defined and not fused to the peduncle; Uropod I without robust seta on peduncle.
*Parhyale explorator*	Parhyale ? iwasai (Shoemaker, 1956)
Uropod III with only apical setae; Propodus of peraeopod VII without setae on posterior margin.	Uropod III with apical and dorsal setae; Propodus of peraeopod VII with setae on posterior margin.
*Parhyale explorator*	*Parhyale hawaiiensis* (Dana, 1853)
Inner ramous of uropod III poorly defined and fused to the peduncle; Propodus of peraeopod VII without setae on posterior margin.	Inner ramous of uropod III well defined and not fused to the peduncle; Propodus of peraeopod VII with setae on posterior margin.
*Parhyale explorator*	*Parhyale penicillata* Shoemaker, 1956
Inner ramous of uropod III poorly defined and fused to the peduncle; Rami of uropods I and II with strong dorsal setae.	Inner ramous of uropod III well defined and not fused to the peduncle; Rami of uropods I and II without strong dorsal setae.
*Parhyale explorator*	*Parhyale fascigera* Stebbing, 1897
Inner ramous of uropod III poorly defined and fused to the peduncle; Rami of uropods I and II with strong dorsal setae.	Inner ramous of uropod III well defined and not fused to the peduncle; Rami of uropods I and II without strong dorsal setae.
*Parhyale explorator*	*Parhyale* of Bulycheva
Inner ramous of uropod III poorly defined and fused to the peduncle; Basipodite of peraeopod VII with rounded posteroventral lobe.	Inner ramous of uropod III well defined and not fused to the peduncle; Basipodite of peraeopod VII without rounded posteroventral lobe.
*Parhyale explorator*	*Parhyale basrensis* Salman, 1986
Inner ramous of uropod III poorly defined and fused to the peduncle; Uropod I with robust seta on peduncle; Propodus of peraeopod VII without setae on posterior margin.	Inner ramous of uropod III well defined and not fused to the peduncle; Uropod I without robust seta on peduncle; Propodus of peraeopod VII with setae on posterior margin.
*Parhyale explorator*	*Parhyale multispinosa* Stock, 1987
Inner ramous of uropod III poorly defined and fused to the peduncle; Propodus of peraeopod VII without setae on posterior margin.	Inner ramous of uropod III well defined and not fused to the peduncle; Propodus of peraeopod VII with setae on posterior margin.

**Figure 5.**
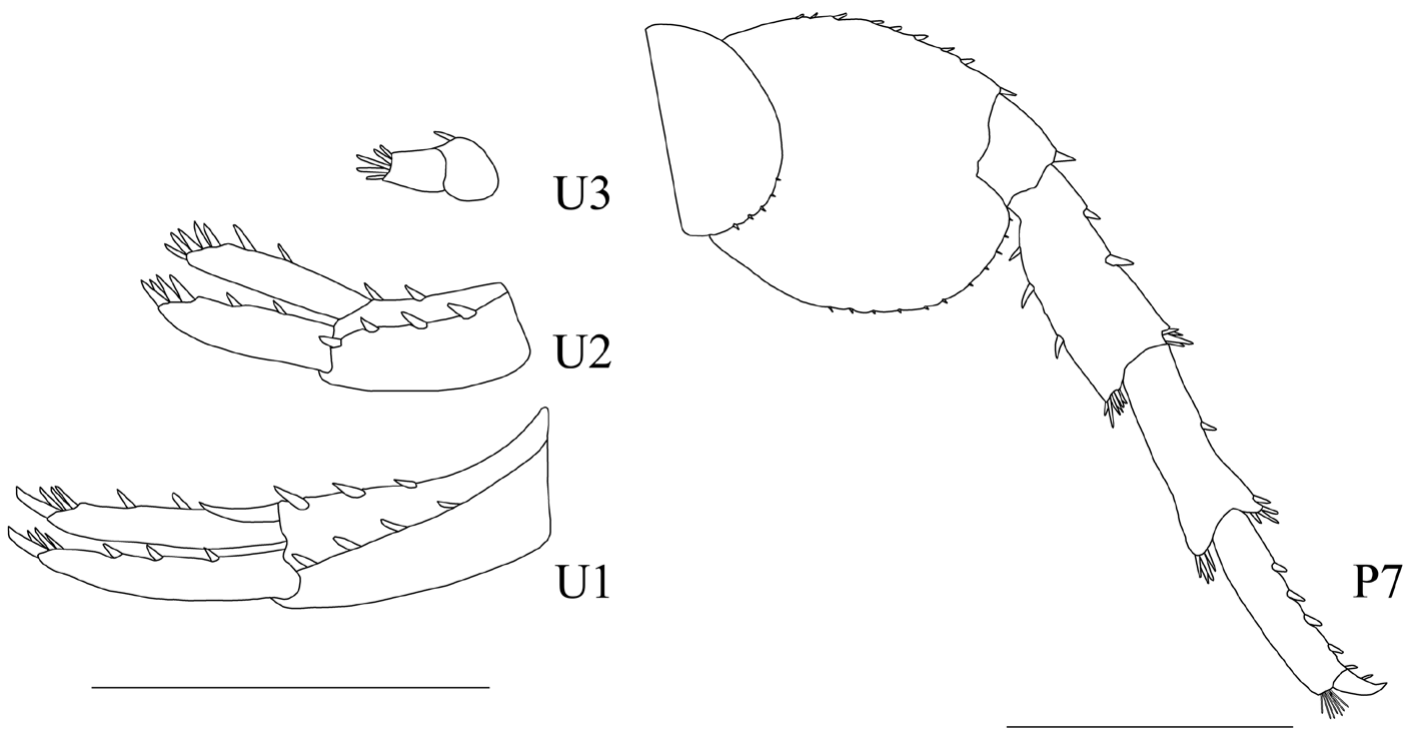
Illustration of male paratype of *Parhyale explorator*, from Ruffo’s collection, uropods I (U1), II (U2), III (U3) and peraeopod VII (P7). Scale bars 1 mm.

The following character states are considered diagnostic of *Ptilohyale explorator*: the arrangement of setae on the uropods and the presence of a strong depression on the posterior margin of the basis of peraeopod VII (Table 2). Here, it has been verified that these characters described in Arresti (1989) did not match with the paratypes (Fig. 5). The setae arrangement on uropod III and the posterior margin of basis of peraeopod VII of the paratypes, on the contrary, matched with specimens recently sampled from the *explorator* type locality and were identified as *Ptilohyale littoralis*. In fact, following the dichotomous key to Hyalidae of Bousfield and Hendrycks (2002), the detailed description of Faasse (2014), the paratypes in Ruffo’s collection, and the present specimens collected in Bay of Arcachon can all be identified as *Ptilohyale littoralis* (Stimpson, 1853). For these reasons, *Ptilohyale explorator* (Arresti, 1989) is proposed as a synonym of *Ptilohyale littoralis* (Stimpson, 1853), which, on base of the Principle of Priority, article 23 of the ICZN Code (Ride Chairman et al. 1999), becomes the valid name of this taxon.

Bousfield (1973) synonymised *Ptilohyale littoralis* with *Ptilohyale plumulosus*, a species distributed along the Pacific coast of North America. This synonymy was subsequently rejected (Bousfield and Hendrycks 2002), thus limiting the distribution of *Ptilohyale littoralis* to the western Atlantic coast of North America (Bousfield and Hendrycks 2002).

Recently, *Ptilohyale littoralis* was declared as a recent alien species spreading along the eastern Atlantic coast since 2009 (Faasse 2014, Spilmont et al. 2016, Marchini and Cardeccia 2017), but this study has shown that the species inhabited the Atlanto-European coast at least since 1985.

Moreover, *Ptilohyale littoralis* was recently recorded along the eastern Pacific coast of North America (Campbell River, Vancouver, Choi et al. 2016; and Puget Sound, Washington State, Heerhartz et al. 2016), suggesting an extension of the species’ range.

The genus *Ptilohyale* has been diagnosed with plumose setae on ventral margins of antenna II that start at the 5^th^ peduncular segment and distomedial robust seta on peduncle of uropod I. Behaviourally, it is described as saltatory and occurring in brackish and estuarine waters (Bousfield and Hendrycks 2002).

*Ptilohyale* is distributed along both the Atlantic and Pacific coasts (Bousfield and Hendrycks 2002, Eun et al. 2014, Faasse 2014, Haswell 1879, Heerhartz et al. 2016, Hiwatari and Kajihara 1981, Hutchings et al. 2013, Kim and Kim 1987, 1991, Macnae 1953, McDermott 1998, Peart 2004, Spilmont et al. 2016, Tsoi and Chu 2005, Tunnell and Withers 2009, Turbeville and Caplins 2010). The genus still includes 12 species in some documents (e.g., Bousfield and Hendrycks 2002, Eun et al. 2014, Horton et al. 2017, Lowry 2010) instead of the eight nominal valid species (Table 4).

**Table 4.** List of *Ptilohyale* species exhibiting diagnostic generic characters, and their distribution.

**Ptilohyale species**	**Distribution**	**Reference**
*Ptilohyale barbicornis* (Hiwatari and Kajihara, 1981)	Japan Sea, Korea and Japan	Hiwatari and Kajihara 1981, Eun et al. 2014
*Ptilohyale bisaeta* (Kim and Kim, 1991)	Japan Sea, Korea	Kim and Kim 1991
*Ptilohyale brevicrus* Eun et al., 2014	Japan Sea, Korea	Eun et al. 2014
*Ptilohyale crassicornis* (Haswell, 1879)*	Tasman Sea, Australia; Yellows and Japan Seas, China and Korea	Haswell 1879, Hiwatari and Kajihara 1981, Kim and Kim 1987, Peart 2004, Tsoi and Chu 2005, Hutchings et al. 2013
*Ptilohyale iole* (JL Barnard, 1970)	Pacific Ocean, Hawaii	Hiwatari and Kajihara 1981
*Ptilohyale littoralis* (Stimpson, 1853)	Atlantic Ocean, France, Netherlands, United States and Canada; Pacific Ocean, Canada	Bousfield and Hendrycks (2002), Choi et al. 2016, Faasse 2014, Spilmont et al. 2016, Heerhartz et al. 2016; this paper;
*Ptilohyale plumulosus* (Stimpson 1857)**	Pacific Ocean, Alaska, Canada and United States	McDermott 1998, Bousfield and Hendrycks 2002, Tunnell and Withers 2009, Turbeville and Caplins 2010, Heerhartz et al. 2016
*Ptilohyale ptilocerus* (Derzhavin, 1937)	Japan Sea, Russia	Hiwatari and Kajihara 1981

* erroneously named *crassicorne* in Bousfield and Hendrycks (2002) instead of *crassicornis*

** erroneously named *plumulosa* in Bousfield and Hendrycks (2002) instead of *plumulosus*

### The Atlantic *Ptilohyale littoralis* vs. the Mediterranean *Parhyale plumicornis*

Due the high connectivity between eastern Atlantic and Mediterranean area, which has already caused a high similarity in the Portuguese and Mediterranean amphipod fauna (Plicanti et al. 2016) it can be supposed that the Atlantic *Ptilohyale littoralis* may have spread into the Mediterranean, or vice versa, *Parhyale plumicornis* into the Atlantic Ocean.

*Parhyale plumicornis* belongs to the Mediterranean fauna and it is the most similar hyalid species to *Ptilohyale littoralis* due to the overlapping morphological and ecological characters such as the brush setae along ventral margin of antennae II (Bakir et al. 2013, Iaciofano and Lo Brutto 2017); and both their presences in the intertidal habitat in slow-drying sediments (Arresti 1989, Bousfield and Hendrycks 2002, Iaciofano and Lo Brutto 2017).

**Figure 6.**
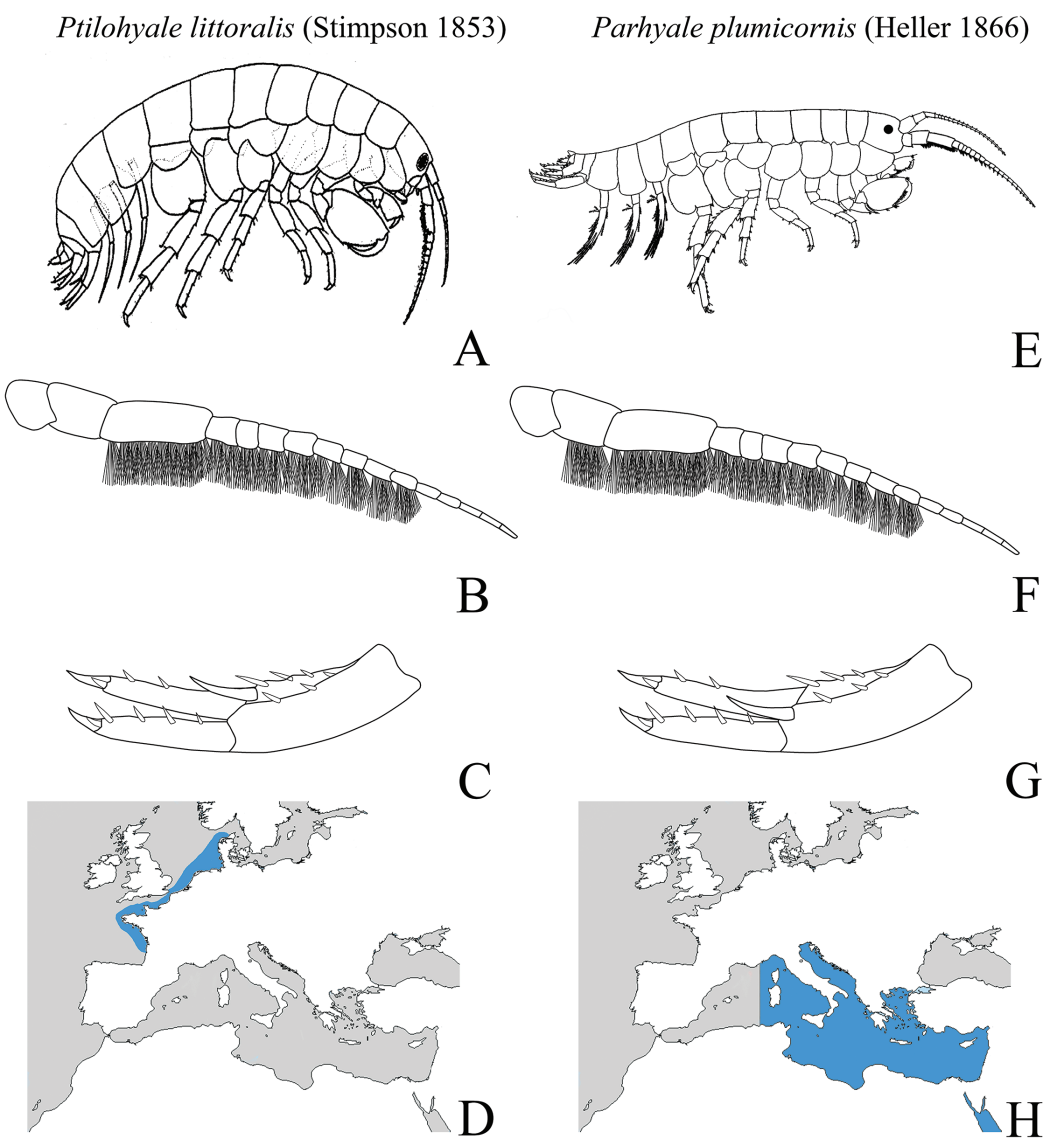
Comparison between *Ptilohyale littoralis* and *Parhyale plumicornis* diagnostic characters and distributions. *Ptilohyale littoralis*: **A** illustration of male (Bousfield and Hendrycks 2002) **B** antenna II male with brush-setae starting at the 5th peduncular segment **C** right uropod I with peduncular distomedial robust seta **D** species distribution along the Atlantic coast. *Parhyale plumicornis*: **E** illustration of male (Iaciofano and Lo Brutto 2017) **F** antenna II male with brush setae starting at the 4^th^ peduncular segment **G** right uropod I with peduncular distolateral robust seta **H** species distribution along the Mediterranean and Red Sea coasts.

Some morphological character states are presented in Fig. 6 as a guide to the correct identifications: *Ptilohyale littoralis* (Fig. 6A) has brush setae on the ventral margin of antenna II that start at the 5^th^ peduncular article (Fig. 6B) and distomedial robust setae on the peduncle of uropod I (Fig. 6C). In contrast, *Parhyale plumicornis* (Fig. 6E) has brush setae on the ventral margin of antenna II that start at the 4^th^ peduncular article (Fig. 6F) and has a distolateral robust seta on the peduncle of uropod II (Fig. 6G).

These two species show a different and non-overlapping distributions: *Ptilohyale littoralis* was recorded along European Atlantic coast (Fig. 6D; Table 4), whereas *Parhyale plumicornis* was recorded along Mediterranean and Red Sea coasts (Fig. 6H; Iaciofano and Lo Brutto 2017 and reference therein). In light of the range extension of *Ptilohyale littoralis* already recorded over long distances, a clear representation of diagnostic character states is needed. *Ptilohyale littoralis* may be invading the Mediterranean Sea where it could probably be a competitor of the Mediterranean endemic *Parhyale plumicornis* as it occupies the same habitat.

## Supplementary Material

XML Treatment for Ptilohyale littoralis
